# The maximum entropy formalism and the idiosyncratic theory of biodiversity

**DOI:** 10.1111/j.1461-0248.2007.01096.x

**Published:** 2007-11

**Authors:** Salvador Pueyo, Fangliang He, Tommaso Zillio

**Affiliations:** 1 Department of Renewable Resources, University of Alberta Edmonton, AB, Canada T6G 2H1; 2 Departament d'Ecologia, Universitat de Barcelona Av. Diagonal 645, 08028 Barcelona, Catalonia, Spain

**Keywords:** Bayesian statistics, diversity patterns, log-normal, log-series, macroecology, maximum entropy formalism, neutral theory, scaling, species abundance distribution, statistical physics

## Abstract

Why does the neutral theory, which is based on unrealistic assumptions, predict diversity patterns so accurately? Answering questions like this requires a radical change in the way we tackle them. The large number of degrees of freedom of ecosystems pose a fundamental obstacle to mechanistic modelling. However, there are tools of statistical physics, such as the maximum entropy formalism (MaxEnt), that allow transcending particular models to simultaneously work with immense families of models with different rules and parameters, sharing only well-established features. We applied MaxEnt allowing species to be ecologically idiosyncratic, instead of constraining them to be equivalent as the neutral theory does. The answer we found is that neutral models are just a subset of the majority of plausible models that lead to the same patterns. Small variations in these patterns naturally lead to the main classical species abundance distributions, which are thus unified in a single framework.

## Introduction

The neutral theory has become one of the pillars of macroecology (Watterson 1974; Caswell 1976; Hubbell 2001; reviews by Chave 2004; Alonso *et al.* 2006; Etienne & Alonso 2006; Hu *et al.* 2006). However, many ecologists doubt that the variety of life can be properly described by a theory based on the assumption that there are no ecological differences among species (according to the standard definition; Hubbell 2001, p. 7; Hu *et al.* 2006). Here, we introduce a radical change of perspective and start from the opposite assumption. We rigorously derive the species abundance distribution (SAD) to be expected when neglecting all ecological similarities among species, instead of neglecting their differences. We call our species ‘idiosyncratic’, in contraposition to the ‘equivalent’ species of the neutral theory. Strikingly, we find exactly the same SAD that is found in simple neutral models: the log-series. We could trace an imaginary line between the extremes of strict neutrality and strict idiosyncrasy and all models on this line would display a log-series, while moderate departures away from the line would lead us to the power law and the skewed log-normal. This suggests a general explanation for virtually all empirical SADs, and, indirectly, for the main types of species–area relationship (SAR).

These findings come after a series of observations in the literature indicating that multiple models, both neutral and non-neutral, lead to similar diversity patterns (McKane *et al.* 2000; Chave *et al.* 2002; McGill 2003a; Mouquet & Loreau 2003; Tilman 2004; Volkov *et al.* 2005; Pueyo 2006a; Nekola & Brown 2007; Zillio & Condit 2007). These patterns transcend particular models and can be best understood by using approaches that also transcend particular models.

The conventional approach to ecological theory is based on mechanistic modelling. The use of mechanistic models often forces us to choose either ignoring the complexity of nature or using so many parameters that hardly any reliability and generality can be expected. However, complexity is not intrinsically incompatible with reliability and generality. If species with diverse ecological features coexist, their singularities may cancel out in community-level measures and give rise to robust regularities. A promising alternative to the analysis of particular models is the study of the statistical properties of large ensembles of complex ecological models, with the aim of identifying such regularities. This is also in the spirit of the log-normal hypothesis, but this hypothesis relies on the precise assumptions of the central limit theorem, and there is no clear justification why these should apply to SADs (Williamson & Gaston 2005). Here we give new results specifically for SADs, using the maximum entropy formalism (MaxEnt) and other related tools, which are well established in statistical physics.

The use of MaxEnt in ecology has a venerable but little known history. Shortly after Jaynes (1957) introduced this method to statistical physics, MacArthur (1960) used a mathematically identical procedure and obtained the ‘broken stick’. However, this is not a realistic SAD. The right solution could not be possibly obtained without the key findings that Jaynes (1968) later added to MaxEnt theory (see ‘The prior distribution’ section). Thereafter, there have been a few isolated attempts to apply MaxEnt to species diversity (Alexeyev & Levich 1997; Levich 2000; Pueyo 2006a; Shipley *et al.* 2006; see also McGill 2006) and related areas (e.g. Luriè & Wagensberg 1983; Wagensberg *et al.* 1991; Hernández *et al.* 2006; Hijmans & Graham 2006; Phillips *et al.* 2006; Pearson *et al.* 2007) but, as far as we know, the way we use it to predict the SAD is entirely new. We compare it with earlier approaches in Appendix A.

**Figure 1:**
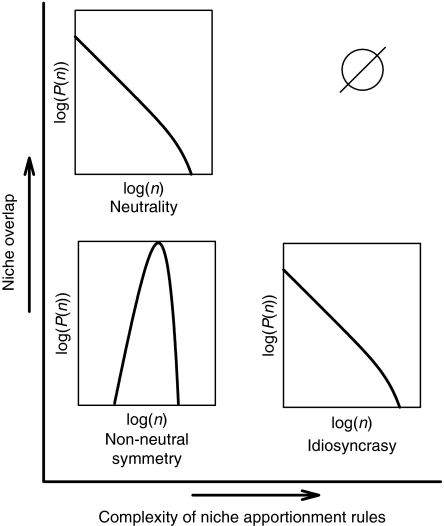
Types of community assemblage. The most extreme option in each of the vertices is indicated and illustrated with a representative example of species abundance distribution (where *n* is abundance and *P*(*n*) is its probability). ‘Niche’ is used in a broad sense, including, e.g. environmental conditions, consumers, infectious diseases and mutualists in addition to resources.

Figure 1 places the idiosyncratic theory in the context of other previous views of community assemblage. The word ‘niche’ is used in a broader sense than usual, including not only resources but also, e.g. environmental conditions, consumers, infectious diseases and mutualists.

Neutral models assume that all species have the same niche, so neutrality corresponds to ‘simple niche apportionment rules’ and ‘high niche overlap’ (Fig. 1). Some parts of Hubbell's (2001) book seem to imply a wider definition of neutrality, but all mathematical results are based on models without niche differentiation (this also applies to the recent extensions of the theory allowing for species-dependent vital rates; Solé*et al.* 2004; Etienne *et al.* 2007; see also Pueyo 2006a, p. 395). The SADs in these models are mainly shaped by a particular mechanism: demographic noise. In principle, a high niche overlap is needed for this mechanism to dominate.

Engen & Lande (1996a) gave some useful tools to predict SADs in more complex models. For example, the inset in the lower left end of Fig. 1 has been obtained with their method, assuming the classical logistic equation plus a moderate environmental noise, but no demographic noise. The absence of demographic noise means that there is no niche overlap and that this model is not neutral. Indeed, the predicted SAD is completely different from that of neutral models. However, we used the same parameter values for all species (*r*, *K* and environmental noise variance *ɛ*^2^), thus introducing a strong symmetry among them. As each species has a different niche, this symmetry does not imply a common resource use or shared interactions of any kind, unlike the main symmetries of neutral models. Therefore, it is a qualitatively different, more abstract type of symmetry, which we call ‘non-neutral symmetry’. The inset in Fig. 1 is one of the simplest examples, but we could design a non-neutral symmetric model for any conceivable SAD. The set of niche apportionment models in Tokeshi (1990; e.g. dominance pre-emption or dominance decay), in which a fix and simple rule is sequentially applied to each of the species in the community, are also non-neutral symmetric models.

Idiosyncrasy is defined by the non-existence of symmetries, either of the neutral or the non-neutral type. Each species is ‘idiosyncratic’ because it is fundamentally different from any other species. Engen & Lande (1996b) gave an important step to idiosyncrasy by extending their equations to sets of species with heterogeneous parameter values, which were assigned at random. However, this method does not necessarily give a fully idiosyncratic SAD. For example, if we used a logistic with *K* following a Gaussian distribution of parameters *μ*_*K*_ and *σ*_*K*_, and applied a similar criterion to *r* and *ɛ*, we would still be assuming particular values for {*μ*_*K*_, *σ*_*K*_, *μ*_*r *_, *σ*_*r* _, *μ*_*ɛ*_, *σ*_*ɛ*_}, and also ignoring possible deviations from the logistic equation, so we would have a residual of non-neutral symmetry. In this paper, we derive the SAD that results from randomness in a more fundamental sense, free of any such residual.

The SAD gives the probability that an unspecified species will have some given abundance *n*. It has two components:

1. The probability for a species chosen at random to display some given ecological features.
2. The probability that a species with some given ecological features has abundance *n*.

By assuming that all species are ecologically equivalent, the neutral theory assumes minimum variability in the first component and maximum in the second. The idiosyncratic theory assumes maximum variability in the first, and either small or large variability in the second. The net result is maximum variability in species abundances in both theories, for completely different reasons.

## The idiosyncratic species abundance distribution

### General setting

Let species *i* have abundance *n*_*i*_, for *i* = 1 to *S*. The probability of the array {*n*_1_,*n*_2_,…,*n*_*S*_} is

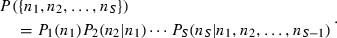
(1)
Assume that each species is ecologically idiosyncratic. If we knew the identity of species 1, we could perhaps predict *n*_1_ with a small error. However, if species 1 is not specified, we do not know which of an infinite set of possible models {*m*_1_,*m*_2_,…,*m*_*W*_}, *W*→∞, best describes its ecological features (these species-level models constitute potential ‘modules’ for the community-level model). Following the criteria in the next section, we can properly define the set of models to ensure that they are equiprobable, with 
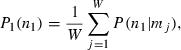
(2)and that the mixture in eqn 2 converges to some well-defined distribution 
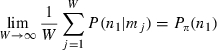
(3)(e.g. Allen *et al.* 2001), analogously to the usual convergence of sums of variables to the Gaussian distribution. For species 2, 
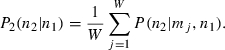
(4)As we know neither the identity of species 2 nor of species 1, and each model will predict a different interaction between them, the fact of knowing *n*_1_ does not reduce the uncertainty about *n*_2_, so we just have a repetition of the same problem in different terms, and eqn 4 will lead to the same limiting distribution as eqn 2: 
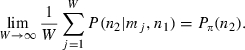
(5)The same reasoning applies to each of the remaining species. Therefore, eqn 1 becomes 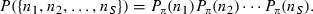
(6)

Abundances enter eqn 6 as independent and identically distributed variables. However, the way this equation was derived makes clear that, in this case, ‘identically distributed’ does not imply ‘ecologically equivalent’ and ‘independent’ does not imply ‘with no ecological interaction’. These properties exclusively hold in the process of predicting the SAD. By contrast, the abundance distribution of a given species in different moments of time will not obey *P*_*π*_: it will depend on its own model *m*_*j*_ (which is different for each species and is assigned only once) and on the interactions with other species. This is a key difference with the neutral theory, which assumes that the abundance distribution of any particular species in different moments of time is identical to the SAD.

In information theoretic terms, each of the models {*m*_1_,*m*_2_,…,*m*_*W*_} contains some amount of information. For example, we could roughly measure this amount by counting the number of words that we need to describe the assumptions of the model. Informally, we could say that, when we perform the linear combination in eqns 3 and 5, the bits of information that are different in different models cancel out. If we include all conceivable models, the resulting distribution *P*_*π*_ will be completely void of information (any bit of information surviving the linear combination would mean some ecological feature that systematically appears in many different species and that needs explanation). This will be the criterion that we will use to find *P*_*π*_.

However, *P*_*π*_ will not be our final result. As we have established no constraint on *P*_*π*_, we could end up with a physically impossible outcome, such as infinite abundances. Therefore, we will transform *P*_*π*_ into another distribution *P*, which will no longer be void of information, but will only include the minimum information for the SAD to be physically meaningful. Strictly speaking, we only use MaxEnt in this last transformation, while the choice of *P*_*π*_ is a previous unavoidable step to apply it. The distribution *P*_*π*_ is called ‘prior distribution’.

### The prior distribution

In eqns 3 and 5, *P*_*π*_ arises by assuming that all models are equally probable. However, equiprobability is ill defined in this case, because there are different criteria to describe and distinguish among different models, which will lead to different asymptotic distributions. This indetermination arises even in much simpler cases, such as the well-known Bertrand's problem of drawing a straight line ‘at random’ intersecting a circle, which can be carried out in different ways depending on different criteria of ‘randomness’. However, Jaynes gave a solution to Bertrand's (Jaynes 1973) and other comparable problems (Jaynes 1968).

Above, we advanced that *P*_*π*_ should be void of information. This is essentially the postulate that Jaynes established in the problems that he treated, and we abide by it. However, the information content of *P*_*π*_ cannot be evaluated by purely mathematical means. We also have to take into account the physical nature of the variable under study. Once we know its nature, we can design one or several mathematical transformations of the variable, such that, if the statistical distribution changes as a result of the transformations, we can say that it contains information. Jaynes’ method, which we call ‘invariance under transformations’, consists of seeking the distribution that does not change under the selected transformations.

We first illustrate this method with a variable that is more intuitive than abundance: spatial position. For example, if an image of the lizard *Podarcis lilfordi* subsp. *brauni* Müller appears in a picture, it is almost sure that it was taken in the 58-ha islet of Colom, off the Mediterranean island of Minorca. Therefore, *P. l. brauni* gives much information about the spatial position of the photographer. An image of the common cockroach *Blatta orientalis* L. would give much less information. For a distribution to give no information about spatial position, it should remain invariant when changing the centre of coordinates (which can be identified with the position of the photographer). This is not the case of *P. l. brauni*: the statistical distribution of the spatial coordinates of the individual lizards will be very different if we set the centre of coordinates in the middle of Colom or 10 km north of the islet. Only the uniform distribution is invariant under this transformation. Therefore, the correct prior distribution for spatial positions is the uniform (for example, this is the distribution of gas molecules in some conditions, but it is not the distribution of *P. l. brauni* because the positions of these lizards do contain information about the ecology and history of the subspecies). Common cockroaches do not have a uniform distribution, but their world distribution is much closer to uniform than *P. l. brauni*’s.

In the case of abundances, we should find a prior *P*_*π*_ such that the abundance of an unspecified species gives no information about any abiotic or biotic factor, because the different responses expected from each of the models in eqns 2–5 should cancel out. While the uniform is the appropriate prior for spatial positions, it is not appropriate for abundances. This assertion is not superfluous. Most authors use the uniform as a standard prior distribution when applying MaxEnt, without a clear justification. Specifically, MacArthur (1960) used it for SADs (but this was before Jaynes’ 1968 paper about prior distributions).

As an example (with some simplifications), if SADs were uniform, the set of abundances of different species of coccolithophores in 100 L of sea water could look like {1 × 10^6^, 3 × 10^6^, 3 × 10^6^, 6 × 10^6^, 8 × 10^6^, 9 × 10^6^}. Without loss of generality, assume that their spatial arrangement is random. Then, in 10 L, we would find a set of abundances close to {1 × 10^5^, 3 × 10^5^, 3 × 10^5^, 6 × 10^5^, 8 × 10^5^, 9 × 10^5^}, and in 1 L we would find something like {1 × 10^4^, 3 × 10^4^, 3 × 10^4^, 6 × 10^4^, 8 × 10^4^, 9 × 10^4^}. Therefore, if SADs were uniform, the abundance of an unspecified species would encode much information on the sampled volume. An abundance of the order of 10^5^ cells would suggest a water volume of the order of 10 L. In nature, such a reliable inference is not possible without knowing the identity of the species. In NW Mediterranean, 10^5^ cells of *Emiliania huxleyi* (Lohmann) Hay and Mohler would suggest a volume of about 30 L, while 10^5^ cells of *Pontosphaera discopora* Schiller would suggest a volume of about 10^4^ L (figures estimated by simple extrapolation from Margalef 1994). At least in this particular aspect, natural SADs contain much less information than the uniform distribution. It follows that the uniform cannot be the uninformative prior.

The mathematical transformation that we will use to choose the prior distribution *P*_*π*_ will be the change in volume or area. This does not imply that invariance in relation to spatial scale is more important than invariance in relation to other abiotic or biotic factors. Invariance in relation to any other factor is a necessary condition for a correct prior distribution, as scale invariance is. However, we found no other factor that allows us to discriminate among abundance distributions in terms of information content. It should also be made clear that a scale-invariant prior SAD does not imply that individual species are also scale invariant: species can have characteristic scales, but these should be different for different species.

In principle, when seeking the prior distribution of abundances, we should assume a random placement of organisms (which results from sampling the uniform prior distribution of spatial positions). However, our results are extremely insensitive to spatial arrangement. If the individuals of a species are randomly located in a large area, the abundance of this species in small sections of this area will follow a Poisson distribution. More generally, ecologists use the negative binomial to fit the spatial abundance distribution of particular species. The smaller the parameter *k* of this distribution, the more clumped the arrangement of the species. The Poisson is a particular case of the negative binomial, with *k*→∞. Our results apply whenever the spatial abundance distribution is a negative binomial, regardless of *k*, and even if different species have different *k*, regardless of the statistical distribution of *k*.

The prior distribution *P*_*π*_ that we find is 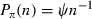
(7)(see Appendix B). Only for this distribution spatial scale does not affect abundance, and abundance gives no information about spatial scale. Equation 7 is equivalent to a uniform distribution of log (*n*). This result means that, if we know nothing about a species, we should consider all orders of magnitude of its abundance [log (*n*)] equally likely. This is the discrete version of Jeffreys’ prior (Jaynes 1968), which is often used for continuous variables in the Bayesian statistical literature. In the ecological literature, this distribution is called geometric series and was probably the first SAD ever proposed (Motomura 1932, quoted by May 1975).

A kind of scale invariance had already been found for some SADs (May 1975; Dewdney 1998; Etienne & Alonso 2005). However, it was a weaker form, because only the type of equation was preserved, but not the numerical values of the probability, as needed for a prior distribution and satisfied by eqn 7.

Although eqn 7 is scale invariant under extremely wide assumptions, this property could be lost in some situations: for some types of systematic relationship between abundance and *k* (we assumed none), and for non-trivial spatial arrangements that cannot be modelled with a negative binomial. This does not affect the status of the geometric series as the prior distribution for abundances (because its invariance for a random arrangement is a sufficient condition). However, in these cases the posterior distribution might contain information about spatial arrangement, which will have to be incorporated in a later stage. In the section ‘Relaxing assumptions: from the log-series to the log-normal’ we give an example.

### MaxEnt gives the log-series distribution

Once we have the prior distribution *P*_*π*_, which contains no information, MaxEnt allows us to find the posterior distribution *P* that incorporates some given information. In this case, we only introduce the minimum information for the SAD to be physically meaningful: it should be a proper distribution and the mean abundance 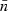 should be finite, which is not the case for eqn 7. While the application of the principle of group invariance is case specific, MaxEnt equations are general. However, we give a complete derivation of these equations for a better understanding of their meaning.

In eqn 6, we sequentially assign an abundance *n* to each of *S* species. As we do not specify the identities of the species, the set {*n*_1_,*n*_2_,…,*n*_*S*_} can be alternatively expressed as {*s*_1_,*s*_2_,…,*s*_∞_}, where *s*_*n*_ is the number of species of abundance *n* (*s*_1_ singletons, *s*_2_ doubletons, etc.). It follows from eqn 6 that the probability *P*({*s*_*n*_}) of each set of species abundances {*s*_*n*_} will follow the multinomial distribution 
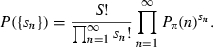
(8)

By definition, the most likely {*s*_*n*_} is the set that maximizes *P*({*s*_*n*_}). In the simplest case, this set satisfies *s*_*n*_≈*SP*_*π*_(*n*). However, this solution may violate some constraints that we know to hold. For example, the sum 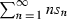 might exceed the total community size. MaxEnt finds the set {*s*_*n*_}, among the sets that satisfy all of the constraints, that maximizes *P*({*s*_*n*_}). The result is expressed as a new probability distribution: *P*(*n*) = *s*_*n*_/*S*.

Maximizing *P*({*s*_*n*_}) in eqn 8 is the same as maximizing 
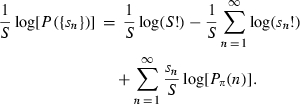
For large *x*, we can use Stirling's approximation 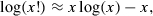
(9)so we have to maximize 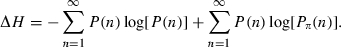
(10)The first of the right-hand side terms in eqn 10 is called ‘entropy’ in statistical physics and information theory (Shannon 1948). In the simplest case of a uniform *P*_*π*_ (Jaynes 1957), maximizing Δ*H* reduces to maximizing entropy *H*; hence, the name of ‘maximum entropy formalism’. However, here we are interested in the general case of maximizing the ‘relative entropy’Δ*H* (Kullback 1959). Often, a constraint *j* can be expressed as a function *h*_*j*_ and a constant *z*_*j*_ as follows: 
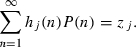
(11)By the definition of probability, a universal constraint for all proper distributions is that the sum of probabilities must be one. This is expressed as *h*_0_(*n*) = 1 and *z*_0_ = 1. We also impose a finite mean abundance as a constraint, so *h*_1_(*n*) = *n* and 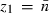.

The distribution {*P*(*n*)} that maximizes Δ*H* while satisfying *J* constraints can be readily found using Lagrange's operator: 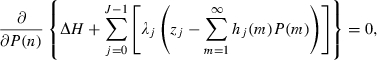
(12)where Δ*H* is defined as in eqn 10, and {*λ*_*j*_} is a set of unknown constants.

The solution of eqn 12 is 
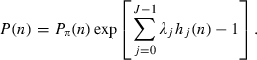
(13)The constants {*λ*_*j*_} can be found by combining eqns 11 and 13.

In the case of the idiosyncratic theory, *P*_*π*_ obeys eqn 7. We introduce eqn 7 into eqn 13, with two constraints (*J* = 2): *h*_0_ = 1 and *h*_1_ = *n*. The result is the classical log-series species-abundance distribution 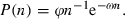
(14)The parameters ϕ and *ω* can be calculated from 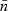 by solving the equations (Fisher *et al.* 1943)
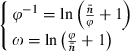


In principle, the exponential decay term in eqn 14 should be considered a good approximation but not the exact, because of our use of Stirling's approximation (eqn 9). On the other hand, MaxEnt equations become exact if, instead of using our probabilistic criterion (which is a generalization of Wallis’; Jaynes 2003, p. 351), they are derived axiomatically (Shore & Johnson 1980). This is a complex technical point which we do not discuss here.

## Relaxing assumptions: from the log-series to the log-normal

We have found the log-series distribution for a ‘hypercomplex’ community, in which each species is completely different from any other species. This is an unrealistic extreme, like complete ecological equivalence. Figure 1 suggests two different ways to decrease complexity, by moving closer to either neutral models or non-neutral symmetric models.

The log-series is well known to be predicted by the type of simple neutral models reviewed by Watterson (1974), and also used by Hubbell (2001) for meta-communities. Strictly equivalent species contain no effective information (in contrast to idiosyncratic species) and their SAD is also uninformative (like the SAD of idiosyncratic species). We could conceptualize the path from neutrality to idiosyncrasy as follows: starting from strictly neutral species with no bit of information, we progressively add bits, which are different for different species and cancel out in the abundance distribution of unspecified species (i.e. the SAD). The abundance distributions of specified species progressively diverge, but the SAD remains invariant.

However, we expect a deviation from the log-series if a part of the bits follow some regularity that prevents their cancellation. This is what we call non-neutral symmetry (see Introduction). The type of modification of the SAD will be different for different types of symmetry, but it may not be possible to discriminate among different types if the modification is modest. Pueyo (2006a) applied Taylor series expansion and found that small deviations from a log-series give a bounded power law, and moderate deviations give a bounded log-normal. ‘Bounded’ means that, above some abundance, close to the upper end of the distribution, probabilities decay faster than expected from a standard power law or a standard log-normal. The equations of MaxEnt allow us to concisely describe the terms in the Taylor series as constraints on the distribution. Nevertheless, as we have not established these constraints *a priori*, our ultimate reason to expect these modifications is the Taylor series and not MaxEnt.

An SAD deviating from the log-series tells us that the constraint on mean abundance is not the only reason why different orders of magnitude of the abundance [log (*n*)] are not equally frequent, as we would expect from the prior distribution (eqn 7). A bounded power law with an exponent slightly different from one indicates that there are some mechanisms causing a slight change in the mean of log (*n*). When this happens, we should modify the idiosyncratic theory by including a term *h*_2_(*n*) = log (*n*) in eqn 13, thus obtaining: 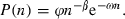
(15)

A bounded log-normal indicates that there are mechanisms causing a slight decrease in the variance of log (*n*), as abundances slightly cluster around a characteristic scale. We introduce *h*_2_(*n*) = log (*n*) and *h*_3_(*n*) = [ log (*n*)]^2^ in eqn 13 and find: 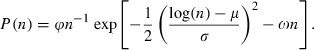
(16)

The normalization constant ϕ in eqns 14–16 can be calculated from the other parameters. Equation 14 is a particular case of eqn 15, for *β* = 1. On its turn, eqn 15 is a particular case of eqn 16, for *σ*→∞ and *μ* = (1 − *β*)*σ*^2^. The standard log-normal is eqn 16 with *ω* = 0, i.e. without an explicit constraint on mean abundance. However, a positive *ω* would account for the seeming ‘left skewness’ that is often found when fitting the log-normal to empirical data sets, as suggested by Pueyo (2006a). This interpretation agrees with the empirical observations by Williamson & Gaston (2005).

There is a non-neutral symmetric feature so common that can be considered trivial: sexual reproduction. It is non-neutral because the ‘resource’ (potential mates) differs according to species, and symmetric because all species display a similar relation between species abundance and ‘resource’ abundance. In sets of species with reproduction largely or exclusively sexual, we expect the SAD to converge to a bounded log-normal for large sizes. If the log-series was indefinitely extrapolatable, most species would have a single representative in the whole world, but such species would not be viable (Allee effect). Therefore, we eventually come back to the log-normal, but it is no longer symmetric and the reasons to expect it are no longer based on a simplistic application of the central limit theorem.

Besides sexual reproduction, other mechanisms could favour a humped SAD. The Janzen–Connell effect might have this effect (Volkov *et al.* 2005), and it is empirically supported (Wills *et al.* 2006).

Hubbell's community model is a special case. In spite of being neutral, it also deviates from the log-series and gives a log-normal-like SAD. This SAD has been analytically derived (Vallade & Houchmandzadeh 2003; Volkov *et al.* 2003; Etienne & Olff 2004; McKane *et al.* 2004; Etienne 2005; Etienne & Alonso 2005) and does not strictly coincide with eqn 16. In practice, however, data sets that are well fitted by this SAD (Volkov *et al.* 2003) are also well fitted by the log-normal (McGill 2003b; Pueyo 2006a) and even by eqn 15 (Azaele *et al.* 2006). In this model, the landscape is assumed to be divided into a set of patches, with dispersal limitation but only at the scale of the patch (distances within or between patches play no role). This precise spatial scale, combined with a migration parameter equal for all species, translates into a characteristic scale in the SAD. Therefore, the SAD contains information that results from a non-trivial type of spatial arrangement. This mechanism is not necessarily more general than the other mechanisms mentioned above.

## Discussion

The log-series is the ‘maximum entropy’ SAD (for a properly defined ‘relative entropy’, eqn 10), and slight-to-moderate decreases in entropy are expected to give the power law and a skewed log-normal-like distribution. These results cover virtually all empirical SADs. In particular, the log-series was one of the first SADs ever fitted to empirical data (Fisher *et al.* 1943), which consisted of large samples of moths. More recently, it has been shown to very well describe a data set of 10^5^ Mediterranean marine diatoms (Pueyo 2006a). This means that Mediterranean diatoms have the SAD that we would consider to be the most likely even if we knew nothing about diatoms, just from first principles. The abundance distribution of the 107 species in this data set can be predicted just from the total number of species and individuals, with no significant error.

The high entropy of SADs can result from species heterogeneity (the idiosyncratic theory), from demographic noise (the neutral theory), or, most likely, from a combination of both, making both theoretical approaches necessary for a balanced understanding of nature. The SAD alone gives no information about the relative importance of these two components. However, analysing spatio-temporal data of tropical butterflies, Engen *et al.* (2002) estimated that demographic noise only contributes about 15% of the variability in abundances, which would suggest that idiosyncratic effects are more important than neutral effects. Even for the tropical forest trees in Barro Colorado (Panama), which constitute the main case study of the neutral theory, Hubbell *et al.* (2001) and Ahumada *et al.* (2004) gave convincing evidence that the abundances of different species are separately regulated (see also John *et al.* 2007).

The predictions of the neutral theory and the idiosyncratic theory coincide in terms of patterns but strongly differ in terms of function. If we describe the ecological community as a channel of information (Margalef 1968), the capacity of the channel is the same regardless of the degree of ecological similarity among species, but the use of this capacity is minimal for strictly equivalent species and maximal for strictly idiosyncratic species. For the first, diversity has no effect on stability, because they are functionally a single species, while, for idiosyncratic species, we should in principle expect diversity to increase stability at the limit of a large number of species, because of the averaging effect (Doak *et al.* 1998). Similarly, the extinction rates that the neutral theory predicts have no value for idiosyncratic communities, where extinctions are not a simple consequence of ecological drift.

We have shown that common shapes of SADs can be predicted from extremely general assumptions. This conclusion is extensive to common shapes of SARs, because these shapes are mathematically related to the SADs we found (Pueyo 2006b). We expect more findings to follow, because we think we have correctly identified the prior distribution (eqn 7), which is the Rosetta Stone that allows translating concepts between statistical physics and macroecology.

Even more generally, we hope to have shown that sometimes science can progress without the need of assuming that nature is less complex than it actually is. Of course, there are some simplifications in our approach, but we have moved close to a full acceptance of the complexity of nature, and simple equations have naturally emerged. If this was not possible, there would be no simple regularity in our complex world.

## Appendix A

Here, we compare our approach to other previous attempts to apply MaxEnt to species diversity.

### MacArthur (1960)

MacArthur applied a mathematical procedure identical to MaxEnt to predict an SAD, a few years after Jaynes (1957) introduced MaxEnt to statistical physics. He did not quote Jaynes and might not have known his work. In fact, earlier authors such as Boltzmann and Gibbs had already used similar equations long before Jaynes, whose main contribution was to justify them in terms of information theory. MacArthur did not depart from information theory: he used this formalism to find the distribution that would result from breaking a stick at randomly chosen points. The stick was a metaphor of niche space. The resulting SAD became widely known, with the name of the ‘broken stick distribution’, but it bears little resemblance to empirical SADs. It is close to an exponential distribution

(17)

(Etienne & Olff 2005 gave a more exact form of this SAD). The difference between eqn 17 and our eqn 14 is due to the fact that, instead of explicitly seeking the uninformative prior distribution as we did, MacArthur implicitly assumed a uniform prior

(18)

(introducing eqn 18 into eqn 13 in the main text with the same constraints that we use [*J* = 2, *h*_0_ = 1, *h*_1_ = *n*], eqn 17 is found). This is hardly surprising, as MacArthur published his paper before Jaynes (1968) developed the criteria to choose prior distributions. MacArthur's work inspired the non-neutral symmetric models in Tokeshi (1990) but, as far as we know, his use of MaxEnt had no continuity in the following decades.

### Alexeyev & Levich (1997) and Levich (2000)

These authors used MaxEnt to predict the SAD, and also the abundance of particular species as a function of their resource use. The main difference with our approach is that, instead of maximizing the relative entropy of the species in terms of abundance (eqn 10) as we do, they maximized the entropy of the individuals in terms of species identity, i.e. they maximized the Shannon–Wiener diversity index (introduced by Margalef 1956):

(19)

where *n*_*i*_ is the abundance of species *i*. This approach implies a sharply peaked prior SAD, completely different from either the uniform or the geometric series. We consider that, in terms of SAD, our approach is a step forward in relation to Alexeyev and Levich's, but that their approach is entirely correct in terms of predicting the abundance of particular species.

Our application of MaxEnt is based on the assumption that the probability for an unspecified species to have abundance *n* is independent of how many other species have abundance *n*, except for a few constraints. Alexeyev and Levich's approach implies that the probability for an unspecified individual to belong to species *i* is independent of how many other individuals belong to that species, except for a few constraints. However, for elementary biological reasons, this probability does depend on the number of other individuals in the species, according to a set of rules that will be approximately the same for all of the individuals in the same species. The best illustration is the very fact that organisms are grouped in species, instead of being uniformly scattered in the space of genomic sequences.

On the other hand, we submit that maximizing eqn 19 under constraints is a correct procedure for inferring the abundances of particular species as a function of their ecological features. Even though we know that there are many rare and a few abundant species, we do not know who is rare and who is abundant. Therefore, when asking about particular species, we should give them all the same prior probability, as eqn 19 does. We lose power to predict the SAD but we gain power for species-specific predictions. The SAD will only be approached at the limit, if much information is introduced in the form of constraints.

### Pueyo (2006a)

This author explicitly took into account the problem of the prior distribution and found the log-series SAD. However, instead of using a general criterion to choose the prior based on its information content, as we do in this paper, he derived the prior from the assumption that population dynamics was driven by demographic noise. Therefore, he applied MaxEnt in the context of the neutral theory, and the methods he used to depart from this theory are unrelated to MaxEnt. Here, we have shown that MaxEnt gives the log-series without the need of the assumptions of the neutral theory.

### Shipley *et al.* (2006)

These authors applied MaxEnt by maximizing eqn 19, like Alexeyev & Levich (1997). However, while Alexeyev and Levich used this method to predict the abundance of particular species as a function of their resource use and also to predict the SAD, Shipley *et al.* (2006) limited themselves to predict the abundance of particular species, as a function of several traits of relevance in relation to ecological succession. In principle, this approach is entirely correct, while Alexeyev and Levich's extension to the SAD is unreliable, for the reasons stated above. The success in the prediction of particular abundances using MaxEnt will depend on the amount and type of information available. This goal is different and complementary to the goal of our paper.

## Appendix B

### Theorem 1

Consider an area inhabited by organisms belonging to multiple species, with a random spatial arrangement. Select a fraction *a* ≤ 1 of the total area. Let {*P*(*n*)} be the SAD in *a* (for convenience, here we refer to all abundances *n* ≥ 0, in contrast to the rest of the paper, which only deals with the non-null part of the distribution). The SAD is independent of *a* if and only if it follows the geometric series distribution

(20)

where ψ is a constant.

### Theorem 2

Consider an area inhabited by organisms belonging to multiple species. Select a fraction *a*  1 of the total area. Let {*P*_*a*_(*n*_*a*_)} be the SAD in *a*, for all abundances *n*_*a*_ ≥ 1. Assume that, if a species has abundance *n*_1_ in the larger area, the probability distribution of the abundance of the same species in *a* is a negative binomial

(21)

where *k* is a clumping parameter and Γ is the gamma function (note that the negative binomial is only meaningful for *a*  1). Let different species have either the same or different values of the parameter *k*, according to an arbitrary probability distribution whose density function is {*g*(*k*)}, with *k* independent of *n*_1_ and *n*_*a*_. Then, the SAD {*P*_*a*_(*n*_*a*_}) in the smaller area will be independent of *a* and will equal the SAD {*P*_1_(*n*_1_)} in the larger area if and only if the abundance distribution is the geometric series in eqn 20.

### Proof of Theorem 1

As the spatial arrangement is random, decreasing spatial scale is equivalent to taking a random sample from the larger area. A random sample can be obtained by excluding a series of randomly chosen individuals. Therefore, *P*(*n*) will remain invariant when changing *a* if and only if

(22)

where Δ*P*(*n*) is the change in *P*(*n*) when a randomly chosen individual is excluded. This change follows the master equation

(23)

where *N* is the total number of individuals at a given scale. From eqns 22–23,

(24)

Equation 24 is satisfied if and only if *P*(*n*) has the form in eqn 20.

### Proof of Theorem 2

In the first part of the proof we demonstrate that eqn 20 is a sufficient condition to have the same SAD in *a* and in the larger area, regardless of *a*. In the second part we demonstrate that it is a necessary condition too.

*First part*. From the assumptions of Theorem 2,

(25)

When the SAD in an area is calculated from an SAD in a larger area, with *a*  1, a continuous distribution can be assumed for the SAD in the larger area (Pielou 1977, p. 270). Therefore,

(26)

where

(27)

Replacing eqns 21 and 27 into eqn 26,

(28)

With the change of variables *x*_*k*_ = *n*_1_*a*/(*n*_1_*a* + *k*), and noting that *n*_1_ = *k*/*a* [*x*_*k*_ /(1 − *x*_*k*_)] and d*n*_1_ = *k*/*a* (1 −*x*_*k*_)^−2^d*x*_*k*_, so that d*n*_1_/*n*_1_ = *x*_*k*_^−1^(1 − *x*_*k*_)^−1^d*x*_*k*_, eqn 28 becomes

The integral

is the beta function *B*(*k*,*n*_*a*_), which satisfies (Abramowitz & Stegun 1965, 6.2.1 and 6.2.2)

Therefore,

(29)

As Γ(*n*_*a*_+1) = *n*_*a*_Γ(*n*_*a*_) and  irrespective of *g*, eqn 29 gives rise to eqn 20.

*Second part.* The set {*P*_*a*_(*n*_*a*_)} is independent of *a* if and only if

(30)

From eqns 25 and 30,

The sum  equals *P*_*a*_(*n*_*a*_) in the particular case in which *k* is the same for all species. As we impose that *P*_*a*_(*n*) = *P*_1_(*n*) regardless of *k*, and regardless of the distribution of *k* if *k* takes different values, we can treat this sum as a constant and we obtain

As

because  irrespective of *a*,

(31)

Equation 21 can also be expressed as

(32)

Replacing eqn 32 into eqn 25

(33)

and replacing eqn 32 into eqn 31,

(34)

Comparing with eqn 33, it is readily apparent that eqn 34 is equivalent to

(35)

On its turn, eqn 35 is equivalent to eqn 24. Therefore, the SAD has the form in eqn 20.

### Remark 1

The probabilities in the geometric series (eqn 20) only add up to 1 for *ψ*→0. This type of behaviour is frequent in prior distributions (Jaynes 2003). It is more easily understood by considering that, in a continuous approximation, the geometric series is equivalent to a uniform distribution of log(*n*), with density of probability ψ. As we have established no constraint on *n* yet, log(*n*) has the same odds of taking any value from 0 to infinity. Therefore, the probability of any particular value is vanishingly small (this compares with the probability of the position of a particle for which we have no information: it will be a uniform distribution in the whole universe, with a vanishing probability for any particular position). This effect disappears when the set of abundances is constrained in eqn 13.
